# Dietary calcium status during maternal pregnancy and lactation affects lipid metabolism in mouse offspring

**DOI:** 10.1038/s41598-018-34520-6

**Published:** 2018-11-08

**Authors:** Ping Li, Xuelian Chang, Xiuqin Fan, Chaonan Fan, Tiantian Tang, Rui Wang, Kemin Qi

**Affiliations:** Laboratory of Nutrition, Beijing Pediatric Research Institute, Beijing Children’s Hospital, Capital Medical University, National Center for Children’s Health, Beijing, 100045 China

## Abstract

Calcium plays important roles in lipid metabolism and adipogenesis, but whether its status in early life affects later lipid profiles needs to be clarified. Three to four-week old C57BL/6J female mice were fed with three different reproductive diets containing normal, low (insufficient) and high (excessive) calcium concentrations respectively throughout pregnancy and lactation. At postnatal 21 days, the weaning male and female pups from each group were sacrificed for experiments and the remaining were fed with the normal chow diet for 16 weeks. Meanwhile, some of the weaning female pups from maternal low calcium diet group were fed with the normal calcium, low calcium and high calcium mature diets respectively for 8 weeks. Maternal insufficient or excessive calcium status during pregnancy and lactation programmed an abnormal expression of hepatic and adipose genes (PPAR-γ, C/EBP-α, FABP4, Fasn, UCP2, PPAR-α, HMG-Red1, Acc1, and SREBP-1c) in the offspring and this may lead to dyslipidemia and accumulation of hepatic triglyceride (TG) and total cholesterol (TC) in later life. The effects of maternal calcium status on lipid metabolism were found only in the female adult offspring, but were similar between offspring males and females at postnatal 21 days. Additionally, the dyslipidemia and hepatic lipid accumulation caused by insufficient calcium status in early life may be reversed to some extent by dietary calcium supplementation in later life.

## Introduction

Epidemiological studies show that the prevalence of metabolic syndrome (overweight/obesity, dyslipidemia, hypertension, and hyperglycemia/insulin resistance) has become one of the most important public health problems worldwide during the past several decades. Dyslipidemia, as one of the metabolic risk factors for metabolic syndrome is closely associated with cardiovascular diseases^1,2^. Therefore, improved strategies for its prevention and treatment are urgently needed. Calcium, an essential nutrient for construction of the bone, may play important roles in regulating many other biological activities including muscle constraction, neurotransmitter release, hormone secretion, lipid and glycogen metabolism, adipocyte proliferation and differentiation, as well as body weight balance^3–6^. Major *et al*. found that a low dietary calcium intake led to weight gain by influencing the de novo lipogenesis in adipose tissue which was the early predictor of the obesity^7^. Whereas, increases in dietary calcium intake attenuate diet-induced adiposity by modulating adipocyte intracellular Ca^2+^ and thereby coordinately regulating lipogenesis and lipolysis^8^. Clinical evidence provides consistent data to support calcium increasing whole body fat oxidation and increasing fecal fat excretion^9^. However, progressive increase of serum calcium level is correlated with worsening of lipid profile in men and women undergoing menopause, and calcium supplementation may lead to dyslipidemia^10^.

**Figure 1:**
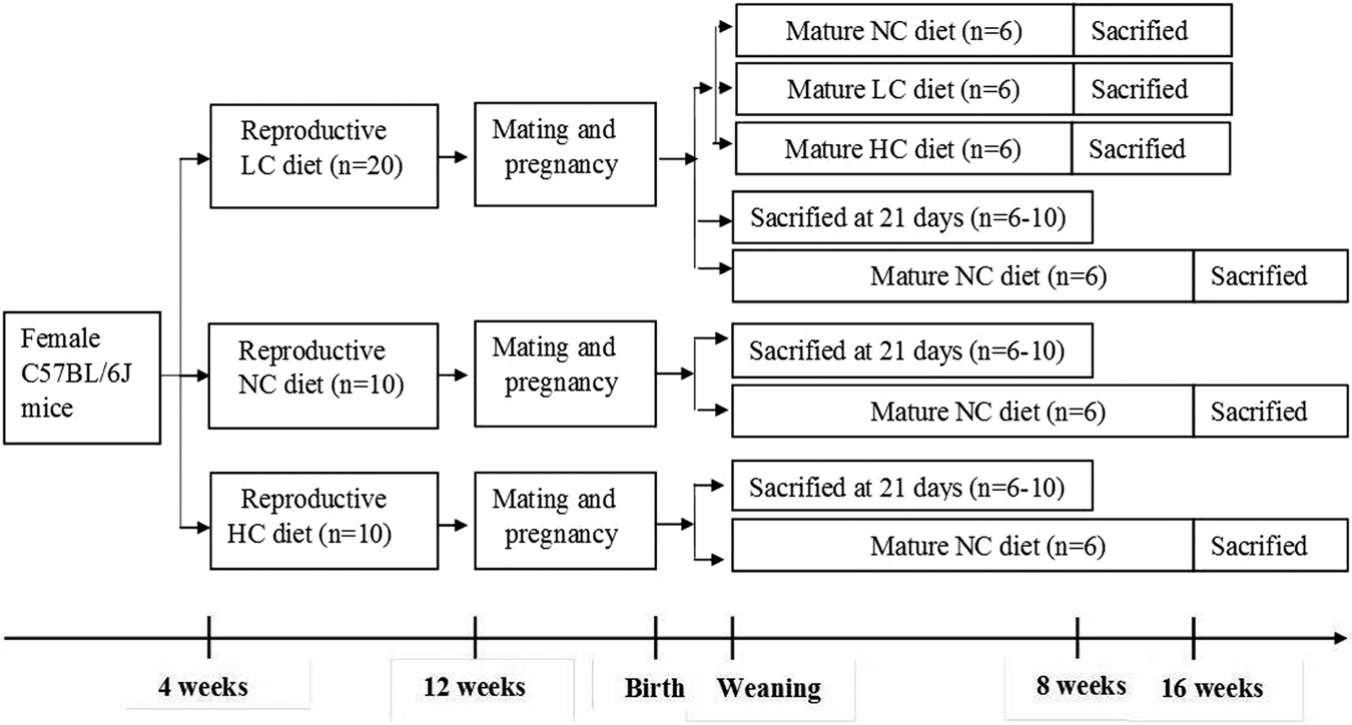
Schematic overview of the study design. LC: diet with low calcium (0.25%, wt/wt); NC: diet with normal calcium (0.70%, wt/wt); HC: diet with high calcium (1.2%, wt/wt).

In recent years, it has been demonstrated that exposure to malnutrition in early life, especially the first 1,000 days, may determine many chronic metabolic disorders including dyslipidemia^11,12^. It is reported that the daily calcium intake is lower than the recommended nutrient intake (RNI) in the pregnant women^13^. In contrast, after birth, the formula-feeding infants or breast-feeding infants with calcium supplementation have more calcium intake than the RNI. This imbalance in calcium intake in early life may have detrimental effects on later health. Thus, we hypothesized that insufficient or excessive intake of calcium in early life might be a risk factor for dyslipidemia in later life. In this study, as shown in Fig. 1, with a mouse model, the effects of low and high calcium status during maternal pregnancy and lactation on lipid metabolism in the offspring with both 21-day and Adult were investigated. Meanwhile, it was determined whether calcium supplementation to the offspring mice from mothers with low calcium status after weaning could lessen the dyslipidemia in later life.

## Results

### Effects of maternal dietary calcium on lipid profiles in the offspring

For the 21-day-old offspring, both in male and female mice, no significant differences were found in body weight, as well as calcium concentrations in plasma and liver among groups with maternal normal calcium (NC), low calcium (LC) and high calcium diet (HC) diets. However, concentrations of triglyceride (TG) and total cholesterol (TC) in the liver and plasma in both maternal LC and HC diet groups were higher than those in maternal NC diet group (Table 1).

**Table 1:** Effects of maternal calcium status on body weight and biochemical parameters in the 21-day offspring mice.

	NC group	LC group	HC group	*F*	*P*
**Male**					
Weight (g)	9.82 ± 1.37	11.58 ± 2.58	9.20 ± 1.97	2.012	0.153
**Calcium**					
Liver (mmol/g)	2.83 ± 1.96	3.40 ± 0.97	3.08 ± 1.08	0.252	0.781
Plasma (mmol/L)	2.24 ± 0.33	2.47 ± 0.24	2.30 ± 0.38	0.811	0.463
**TG (mmol/L)**					
Liver	13.46 ± 2.13	18.44 ± 2.58*	18.11 ± 2.65*	4.672	0.026
Plasma	0.15 ± 0.09	0.36 ± 0.14*	0.40 ± 0.18*	5.154	0.021
**TC (mmol/L)**					
Liver	15.18 ± 3.97	26.93 ± 4.54*	21.22 ± 1.99*	4.721	0.026
Plasma	1.24 ± 0.10	1.80 ± 0.32*	1.78 ± 0.54*	4.476	0.030
**Female**					
Weight (g)	8.36 ± 1.01	9.52 ± 1.10	8.88 ± 1.67	2.011	0.153
**Calcium**					
Liver (mmol/g)	3.33 ± 2.71	2.53 ± 1.42	2.51 ± 1.13	0.367	0.699
Plasma (mmol/L)	2.08 ± 0.20	2.09 ± 0.25	2.19 ± 0.27	0.359	0.704
**TG (mmol/L)**					
Liver	13.14 ± 1.60	16.93 ± 1.24*	16.78 ± 2.57*	4.261	0.034
Plasma	0.45 ± 0.04	0.58 ± 0.07*	0.47 ± 0.09	4.619	0.042
**TC (mmol/L)**					
Liver	14.62 ± 2.66	14.30 ± 1.75	16.43 ± 1.58*	4.612	0.043
Plasma	1.53 ± 0.07	2.14 ± 0.39*	2.41 ± 0.43*	7.248	0.013

Four weeks old C57BL/6J female mice were fed with the NC, LC, HC diets respectively, starting 2 months before mouse conception and continued throughout gestation and lactation. After weaning the pups were sacrificed in a fed state for the experiments. All values were shown as means ± SD. n = 6 in each group for male and n = 10 in each group for female.

*Compared with the NC group, *P* < 0.05.

For the adult offspring, body weight did not change in maternal LC and HC diet groups compared to maternal NC group. Calcium concentrations in the liver were lower in female mice from maternal LC diet group than those in maternal NC diet group, with increased concentrations of TG and TC in the liver or plasma (Table 2).

**Table 2:** Effects of maternal calcium status on body weight and biochemical parameters in the adult offspring mice.

	NC group	LC group	HC group	*F*	*P*
**Male**					
Weight (g)	27.10 ± 1.96	27.08 ± 3.74	27.78 ± 1.50	0.245	0.784
**Calcium**					
Liver (mmol/g)	9.57 ± 2.43	7.61 ± 5.06	9.49 ± 2.31	0.744	0.493
Plasma (mmol/L)	2.75 ± 0.43	3.21 ± 0.33	3.54 ± 0.45	2.615	0.108
**TG (mmol/L)**					
Liver	12.56 ± 4.30	13.50 ± 2.29	9.93 ± 4.72	1.340	0.291
Plasma	0.35 ± 0.12	0.39 ± 0.09	0.40 ± 0.17	0.981	0.398
**TC (mmol/L)**					
Liver	14.20 ± 5.40	15.41 ± 4.02	12.73 ± 2.68	0.620	0.551
Plasma	2.38 ± 0.40	2.63 ± 0.27	2.45 ± 0.63	0.492	0.621
**Female**					
Weight (g)	21.85 ± 1.69	22.05 ± 1.07	22.50 ± 1.50	0.511	0.606
**Calcium**					
Liver (mmol/g)	8.61 ± 3.64	5.39 ± 1.56*	9.62 ± 2.37	6.132	0.012
Plasma (mmol/L)	2.42 ± 0.13	2.99 ± 0.36	3.18 ± 0.67	1.377	0.283
**TG (mmol/L)**					
Liver	15.82 ± 2.37	25.80 ± 8.26*	13.72 ± 4.32	8.090	0.004
Plasma	0.32 ± 0.10	0.49 ± 0.10*	0.39 ± 0.10	4.032	0.040
**TC (mmol/L)**					
Liver	14.09 ± 3.60	18.72 ± 4.67*	12.02 ± 3.37	4.588	0.028
Plasma	3.11 ± 0.83	3.52 ± 1.15	3.42 ± 0.71	0.318	0.732

Four weeks old C57BL/6J female mice were fed with the NC, LC, HC diets respectively, starting 2 months before mouse conception and continued throughout gestation and lactation. After weaning the pups were fed with the normal calcium mature diet for 16 weeks. All values were shown as means ± SD. n = 6 in each group for both the male and female.

*Compared with the NC group, *P* < 0.05.

As was shown in Table 3, dietary calcium supplementation, after weaning for 8 weeks, had a significant impact on hepatic and plasma lipid profiles in offspring mice from maternal LC diet group. Specifically, compared with mice from the maternal LC diet group continually fed with the LC diet after weaning (LC-LC group), TG and TC concentrations in the liver were reduced both in mice from the LC-NC and LC-HC groups with no changes in plasma among these three groups.

**Table 3:** Changes of body weight and biochemical parameters with the calcium-insufficient female offspring by dietary calcium intervention.

	LC-LC group	LC-NC group	LC-HC group	*F*	*P*
Weight (g)	18.29 ± 1.71	17.82 ± 1.52	18.67 ± 0.86	1.006	0.378
**Calcium**					
Liver (mmol/g)	4.28 ± 1.01	5.16 ± 0.63*	7.24 ± 0.61*	9.482	0.002
Plasma (mmol/L)	2.34 ± 0.59	2.54 ± 0.61	2.79 ± 1.06	1.425	0.262
**TG (mmol/L)**					
Liver	28.92 ± 8.74	18.21 ± 5.79*	15.67 ± 4.94*	10.686	0.001
Plasma	0.28 ± 0.12	0.38 ± 0.12	0.29 ± 0.10	1.315	0.298
**TC (mmol/L)**					
Liver	28.14 ± 8.69	17.01 ± 3.23*	14.62 ± 3.08*	6.406	0.005
Plasma	1.88 ± 0.68	1.80 ± 0.61	1.50 ± 0.49	2.424	0.097

Four weeks old C57BL/6J female mice were fed with the LC diet, starting 2 months before mouse conception and continued throughout gestation and lactation. After weaning, female pups were fed with the low calcium (LC-LC), normal calcium (LC-NC) and high calcium (LC-HC) mature diets respectively for 8 weeks. All values were shown as means ± SD. n = 6 in each group.

*Compared with the LC-LC group, *P* < 0.05.

**Figure 2:**
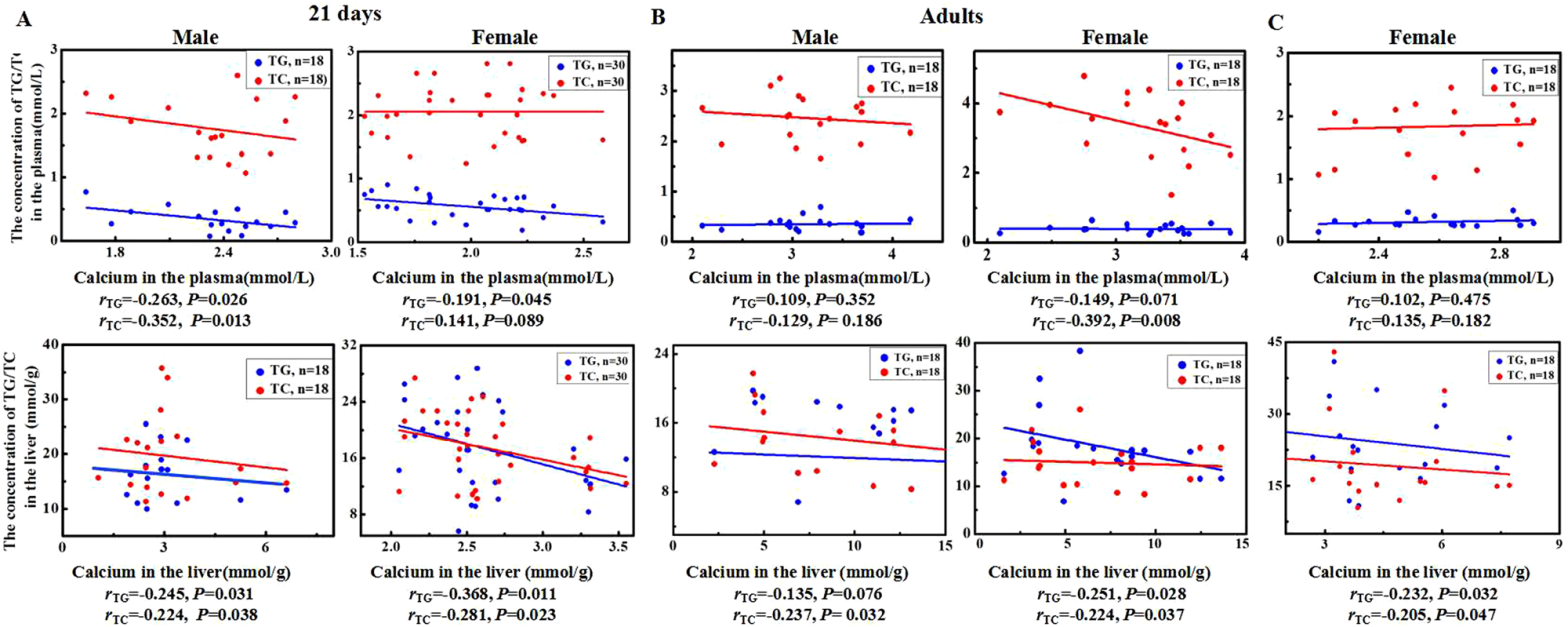
Correlations between calcium concentrations and lipid levels in the plasma and liver in the offspring mice. Four weeks old C57BL/6J female mice were fed with the NC, LC, HC diets respectively, starting 2 months before mouse conception and continued throughout gestation and lactation. After weaning some pups from the three groups (n = 6 in each group for male, and n = 10 in each group for female) were sacrificed in a fed state for the experiments (**A**). Others were fed with the normal calcium mature diet for 16 weeks (n = 6 in each group for both male and female), and sacrificed in a fasted state (**B**). Additionally, some female pups from the LC diet group were fed with the low calcium (LC-LC), normal calcium (LC-NC) and high calcium (LC-HC) mature diets respectively for 8 weeks (n = 6 in each group), and sacrificed in a fasted state (**C**). Calcium, TG and TC were measured in plasma and liver of the offspring mice and the correlations between them were analyzed.

The correlative analysis showed negative associations of calcium with TG or TC levels in plasma or liver in both the male and female offspring at postnatal 21 days, and these associations in the adult offspring were primarily found in the female. In the female offspring with dietary calcium intervention, negative correlations were shown between calcium and TG or TC levels in the liver (Fig. 2).

### Effects of maternal dietary calcium on hepatic and adipose histology in the offspring

**Figure 3:**
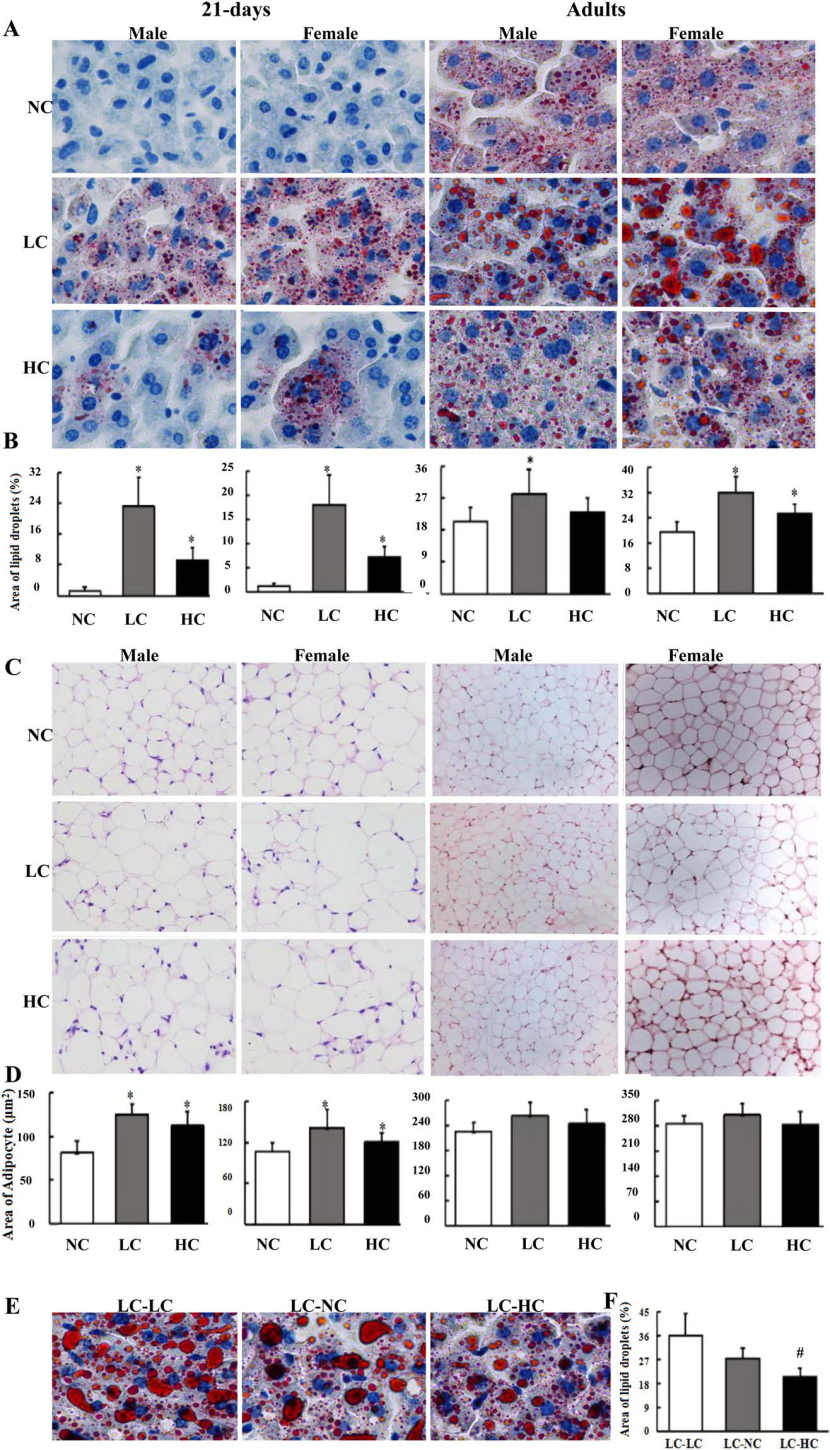
Maternal calcium status affects the hepatic and adipose histology in the offspring mice. The mouse experimental procedure was the same as in Fig. 2, and three samples for each tissue in each group were randomly selected for histological analysis. (**A**) Oil red O staining (×400) of the liver in the offspring; (**C**): hematoxylin/eosin (HE) staining (×200 for 21 days, and ×100 for adults) of the epididymal fat in the offspring. (**E**) Oil red O staining (×400) of the liver in calcium insufficient female offspring with calcium supplementation. Changes in adipocyte size and hepatic lipid droplet quantity in the offspring mice affected by maternal calcium status were shown in (**B**) (21 day); (**D**) (adults) and (**F**) (calcium insufficient female offspring with calcium supplementation). The Image-pro Plus (IPP) was used to quantitatively analyze the size of fat cells in adipose tissues and the amount of lipid droplets in hepatic tissues. *Compared to the NC group, *P* < 0.05; ^#^Compared to the LC-LC group, *P* < 0.05.

As were shown in Fig. 3, at postnatal 21 days or in adults, more lipid droplets in hepatocytes were found in both male and female offsprings from the LC and HC diet groups compared to the NC diet group (Fig. 3A,B). Adipocyte size was larger in 21-day-old offspring from the LC and HC diet groups than that in the NC diet group, whereas no differences were found in the adult offspring among the three groups, either with males or females (Fig. 3C,D). As compared with the LC-LC group, less lipid droplets in the hepatocytes were found in the LC-HC group with a declining trend in the LC-NC group (Fig. 3E,F), indicating that dietary calcium supplementation to weaning mice with low calcium intake during maternal pregnancy and lactation could reverse their dyslipidemia to some extent.

### Effects of maternal dietary calcium on expression of genes related to lipid metabolism in the offspring

**Figure 4:**
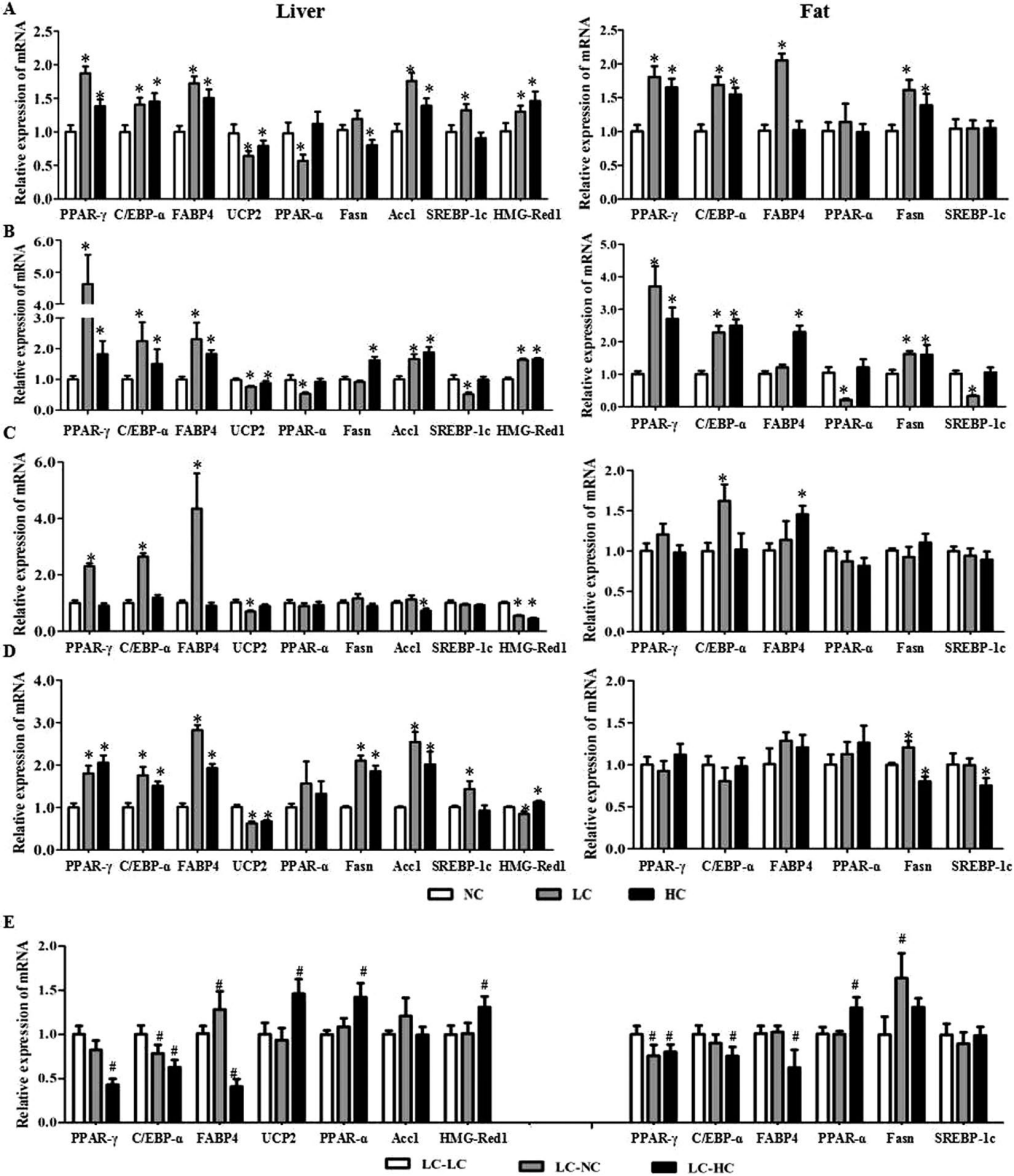
Maternal calcium status affects expressions of genes related to lipid metabolism in the offspring mice. The mouse experimental procedure was the same as in Fig. 2. The mRNA expression of genes PPAR-γ, C/EBP-α, FABP4, UCP2, PPAR-α, Fasn, HMG-Red1, Acc1 and SREBP-1c in the liver or epididymal fat were determined by RT-PCT. (**A**) 21-day male; (**B**) 21-day female; (**C**) adult male; (**D**) adult female; (**E**) calcium insufficient female offspring with calcium supplementation. *Compared to the NC group, *P* < 0.05; ^#^Compared to the LC-LC group, *P* < 0.05.

With the 21-day male offspring, in general, the expressions of uncoupling protein 2 (UCP2) and peroxisome proliferators activated receptor-alpha (PPAR-α) were down-regulated, and peroxisome proliferators activated receptor-gamma (PPAR-γ), CCAAT enhancer binding protein-alpha (C/EBP-α) and fatty acid binding protein 4 (FABP4), fatty acid synthesis (Fasn), acetyl coenzyme A carboxylase 1 (Acc1), hydroxymethylglutaryl-CoA reductase (HMG-Red1) and sterol regulatory element binding protein-1c (SREBP-1c) were up-regulated in the liver from the LC or HC diet groups, compared to the NC diet group (left panel in Fig. 4A). Similar results were found in the female offspring except for the SREBP-1c which was down-regulated in the LC diet group (left panel in Fig. 4B). In the adipose tissue, the LC or HC diet increased expressions of PPAR-γ, C/EBP-α, FABP4 and Fasn in both the male (right panel Fig. 4A) and female (right panel in Fig. 4B) offspring, and only the LC diet reduced expressions of PP AR-α and SREBP-1c in the female offspring (right panel in Fig. 4B).

In the adult offspring, changes in expressions of genes related to lipid metabolism in the female liver from the LC and HC diet groups showed similar profiles with the 21-day-old offspring, as compared to the NC diet group (left panel in Fig. 4D); whereas only UCP2 and HMG-Red1 were down-regulated by the LC or HC diet in the male liver (left panel in Fig. 4C). In the adipose tissue, the LC diet increased the Fasn expression and the HC diet reduced expressions of Fasn and SREBP-1c in the female offspring (right panel in Fig. 4D), with up-regulated expressions of C/EBP-α and FABP4 by the LC and HC diets respectively in the male offspring (right panel in Fig. 4C).

As shown in Fig. 4E, for the offspring from the LC diet group, dietary calcium supplementation with high amount increased expressions of UCP2 and PPAR-α, and decreased those of PPAR-γ, C/EBP-α and FABP4 in the liver, and similarly increased the PPAR-α expression and decreased expressions of PPAR-γ, C/EBP-α and FABP4 in the adipose tissue. However, normal dietary calcium supplementation to the offspring from the LC diet group had less effects on genes’ expression, with reduced C/EBP-α and increased FABP4 in the liver, and increased Fasn in the adipose tissue.

## Discussion

It has been proved that dietary calcium deficiency is closely associated with dyslipidemia and hepatic steatosis, and predisposes for multiple chronic diseases^13–15^. In this study, using mouse models, we found that dietary calcium status in early life affected later lipid metabolism, which might increase risks of lipid related chronic diseases. Insufficient or excessive intake of calcium during maternal pregnancy and lactation increased plasma concentrations of TG and TC, and hepatic lipid accumulation in their offspring at postnatal 21 days, with similar effects on the male and female. The calcium’s effects on the adult offspring became smaller, with increased plasma concentrations of TG and TC, and hepatic lipid accumulation only in the female from the LC diet group. These data indicated that calcium status in early life had a long lasting effect on lipid metabolism especially in the female.

It is agreed that calcium transfer from mother to the fetus is attributable to the placental active transport, resulting in the higher concentration of blood calcium in the fetus than that in the mother. During pregnancy, there is an increase of efficiency in maternal intestinal calcium absorption and bone turnover to meet the basic demand of fetus. However, even with these high rates of absorption, maternal and fetal needs may not be met in women with chronically low calcium consumption^16–18^. Usually, in rodents, diet calcium concentrations ranged from 0.4% to 1.0% promotes mouse normal growth and development, and calcium at a concentration of 0.02% would cause delay in growth. We used the 0.7% calcium diet as normal, which was slightly higher than 0.57% calcium in the breeding AIN-93G diet. Thus, the calcium concentration at 0.25% in the LC diet represented an insufficient level, for typical breeding diets, while its concentration at 1.2% in the HC diet was calcium-excessive^19,20^.

The mechanisms by which calcium affects lipid metabolism have been considered to be related to several pathways. Calcium may combine with fatty acids and bile acids to form insoluble calcium soap in the intestine, leading to more fecal fat excretion^21,22^. Also, calcium supplementation increases intracellular calcium concentration to regulate the activity of Fasn and hormone sensitive lipases, which results in a reduction of fat storage and increases in fat decomposition and oxidation^19^. Dietary calcium supplementation is reported to be able to improve cardiovascular parameters and brown adipose tissue thermogenesis capacity in adult animals that were early overfed during lactation^23^. Studies in mice have shown that an increase in dietary calcium consumption creates a shift in the utilization of energy stores from carbohydrates to fat and a shift in the partitioning of energy from storage (as fat) to expenditure (as heat)^8,23^. Other mechanisms may also be involved, because calcium imbalance may change secretion of parathyroid hormone and 1,25-dihydroxyvitamin D concentration, which regulated adipocyte activity and lipolysis^24,25^.

In our study, in general, the insufficient calcium status during maternal pregnancy and lactation down regulated expressions of the UCP2 and PPAR-α and up regulated those of PPAR-γ, C/EBP-α, FABP4, Fasn, HMG-Red1, Acc1, and SREBP-1c in the liver or adipose tissue of the offspring. UCP2 can be dissipated to uncoupling the respiratory chain and the synthesis of ATP to affect thermogenesis and heat energy metabolism, some researches had proved UCP2 deficiency caused diminished hepatic utilization and fatty acid clearance and thus may lead to liver steatosis^26,27^. PPAR-α and SREBP-1, key transcription factors, play important roles in lipid metabolism. PPAR-α regulates gene expressions involved in FA oxidation, inflammation, oxidative stress, lipid and lipoprotein metabolism. Activation of PPAR-α stimulates beta-oxidation of fatty acids, thereby reducing serum TG level; and reduced PPAR-α expression in liver causes TG storage and steatosis^28,29^. Whereas over expression of SREBP-1c results in increased hepatic synthesis and accumulation of TG^30,31^. Expression of PPAR-γ, C/EBP-α, FABP4, Fasn and Acc1 results in the increased production of TG and adipocyte proliferation and differentiation in mice as well as in human subjects, which further leads to hepatic steatosis^32^. The hepatic TG accumulation in this study may be attributable to the up-regulated expressions of these adipogenesis associated genes with calcium insufficiency. HMG-CoA-Red 1 was a key enzyme of cholesterol synthesis in the liver to inhibit the synthesis of cholesterol and improve the LDL, HDL and other indicators of blood triglyceride^33,34^. Herein, the reduced HMG-Red1 mRNA expression in adult male offspring from the LC and HC groups could not explain the unchanged liver cholesterol level, and the underlying reasons need to be explored further. Nonetheless, increased synthesis of triglyceride and cholesterol and reduced fatty acid oxidation may also be responsible for the dyslipidemia caused by low calcium status in early life.

It is clear that high calcium intakes have the potential effect to cause hypercalcemia, being associated with renal insufficiency, vascular and soft tissue calcification and kidney stones, as well as primary hyperparathyroidism or malignancy^35–37^. In this study, excessive calcium status during maternal pregnancy and lactation was found to worsen expressions of lipid related genes in the liver and adipose tissue and hepatic lipid accumulation in the 21 d or adult offspring mice. In keeping with this, an observational study on a large cohort showed a positive association between serum lipid levels (cholesterol and triglycerides) and serum calcium in men and postmenopause women^10^. It is worthy to note that serum calcium is very tightly regulated and does not fluctuate with changes in dietary intakes; the body uses bone tissue as a reservoir for, and source of calcium, to maintain constant concentrations of calcium in blood, muscle, and intercellular fluids^35–37^. This may explain calcium concentrations changed in the adult liver with calcium supplementation but no changes found in plasma.

Interestingly, the calcium’s effects on lipid metabolism in the adult offspring were gender specific. Low calcium intake during maternal pregnancy and lactation resulted in increased plasma concentrations of TG and TC, and hepatic lipid accumulation in female instead of male. This is consistent with Sharma’s reports showing that chronic maternal calcium deficiency in Wistar rat programs abnormal hepatic gene expression leading to hepatic steatosis in female offspring^38^. Clinical studies demonstrated that calcium intake might have an effect on serum lipid concentrations predominantly in women instead of men^39,40^. However, the mechanism underlying the sexual dimorphic effects of calcium on lipid metabolism were not clearly understood, and needs to be further studied. Furthermore, we examined the interventional effects by calcium supplementation after weaning to the female offspring from the LC diet group, and found that high calcium intake lessened the hepatic lipid storage and disturbed gene expressions.

To note, in this study, we aimed to determine the relations between early calcium status and later lipid metabolism, and found that calcium insufficiency or excess in maternal pregnancy and weaning periods adversely affected lipid metabolism in adults, even if with normal calcium intake later on. In recent years, more and more attention has been paid to understanding the environmental risk factors including unbalanced diet and nutrition in the critical periods of early life for later health and diseases, and one of the underlying mechanisms is involved in epigenetic modification^11,12,41,42^. Recently, a study reported that calcium was able to modify the expression and methylation pattern of genes involved in energy balance in adulthood, including adiponectin, stearoyl-CoA desaturase and Fasn^43^. As well, we have found that insufficient or excessive status of calcium in early life has a long term effect on brain acretion of docosahexaenoic acid mediated by altered DNA methylation and associated expressions of fatty acid desaturases, and also a long lasting adverse effect on gut microbiota (unpublished data), which have been proved to be closely associated with dyslipidemia and related chronic diseases^44,45^. Thus, the effect of calcium on epigenetic modification needs to be clarified in detail.

## Conclusion

Maternal insufficient or excessive calcium status during pregnancy and lactation programmed an abnormal expression of hepatic and adipose genes in the offspring, leading to dyslipidemia and hepatic lipid accumulation. Specifically, maternal calcium primarily affected lipid metabolism in the female adult offspring, with similar effects on lipid metabolism between the male and female 21 day offspring. The dyslipidemia and hepatic lipid accumulation caused by early insufficient calcium status may be reversed to some extent by dietary calcium supplementation in later life.

## Materials and Methods

### Diets

Three types of reproductive diets with normal calcium (NC), low calcium (LC) and high calcium (HC) concentrations (0.70%, 0.25% and 1.20%, wt/wt, respectively) were designed, based on the gestating and growing formula (D10012G with standard of AIN-93 G) from the Research Diets, Inc. (New Brunswick, NJ). Also, mature NC (0.70%, wt/wt) (normal chow diet), LC (0.25%, wt/wt) diet and HC (1.20%, wt/wt) diets were also designed, based on the formula for mature rodents (D10012M) from the Research Diets, Inc. Usually, diet calcium concentrations ranged from 0.4% to 1.0% promotes mouse normal growth and development, and calcium at a concentration of 0.02% would cause delay in growth. We used the 0.7% calcium as normal, which was slightly higher than 0.57% calcium in the breeding AIN-93G diet. Thus, the calcium concentration at 0.25% in the LC diet represented an insufficient level instead of deficient, for typical breeding diets, while its concentration at 1.2% in the HC diet was calcium-excessive^19,20^. These diets were prepared by the Institute of Laboratory Animal Sciences at the Chinese Academy of Medical Sciences and stored at −20 °C before use.

### Animals

Three- to four-week-old C57BL/6J female mice were purchased from SPF Laboratory Animal Technology Co., Ltd (Beijing) and housed at the animal facilities with cycles of air ventilation and free access to water and food in the Laboratory Animal Center of the First Affiliated Hospital of the People’s Liberation Army (PLA) General Hospital of China. The living environment was maintained at a temperature of 22 °C and a humidity of 50% under a 12 hour (h) light 12 h^−1^ dark cycle. All the female mice were randomly divided into three groups and fed with the reproductive NC, LC and HC diets respectively. After 8 weeks of feeding, the female mice in each group were mated with the normal adult male mice and continued on their own diet throughout gestation and lactation. The newborn male and female pups from each group, after weaning at postnatal 21 days, were sacrificed in a fed state for the experiments. The rest of male and female pups from all the three groups were fed with the normal chow diet for 16 weeks. Meanwhile, the female pups from the LC diet group were classified into three groups, and fed with the matue LC diet (LC-LC group), mature NC diet (LC-NC group) and mature HC diet (LC-HC group) respectively for 8 weeks. At the end of study, after 12-h fasting, pups were anesthetized by intraperitoneal injection of Avertin (2,2,2-tribromoethanol) (T-4840-2; Aldrich Chemical, Munich, Bavaria, Germany) (125 mg/kg) and the blood samples were obtained by heart puncture and then sacrificed by injection of an overdose of Avertin (500 mg/kg) and decapitation to minimize suffering. Immediately, the epididymal fat and liver were dissected free of the surrounding tissue, removed, wrapped in aluminum foil and frozen in liquid N_2_. Once an entire group of animals was harvested, the tissues were transferred to −80 °C until the analysis was performed. A schematic overview of the study design is shown in Fig. 1.

All of these animal experiments were performed from 08:00 to 12:00 in accordance with the recommendations in the Guide for the Care and Use of Laboratory Animals of National Administration Regulations on Laboratory Animals of China. The protocol was approved by the Committee on the Ethics of Animal Experiments of the First Affiliated Hospital of PLA General Hospital in China.

### Measurements of biochemical parameters

Calcium concentrations in mouse plasma, liver and epididymal fat were determined by the colorimetric method with the calcium detection kit (cat. no. C004-1, Nanjing Jiancheng Bioengineering Institute, China). Plasma and hepatic TG and TC concentrations were assayed by an an enzymatic procedure with a TG assay kit by GPO-PAP method and a TC assay kit by COD-CE-PAP method respectively (Maccura Biotechnology Co., Ltd, Sichuan, China). The BCA-protein quantification assay kit (P0010S, Beyotime Institute of Biotechnology, Shanghai, China) was used to measure protein contents in hepatic tissues. The data were expressed as mmol/g protein for the liver and mmol/L for the plasma.

### Histological analysis

After the mice were sacrificed, three samples for each tissue in each group were randomly selected for histological analysis. The hepatic and adipose tissues were quickly collected and fixed in 10% buffered formalin. Then the hepatic tissues were stained with Oil red O and examined under light microscope at magnification of 400× to assess morphology and lipid droplets; whereas the adipose tissues were embedded in paraffin and cut into 6 μm sections to be stained with hematoxylin/eosin (HE) and examined under light microscope for morphological assessment. Finally the Image-pro Plus (IPP) was used to quantitatively analyze the size of fat cells in adipose tissues and the amount of lipid droplets in hepatic tissues.

### Gene expression analysis by the RT-PCR

Total RNA was extracted from the liver and epididymal fat using Trizol Reagent (cat. no. 15596-206, Invitrogen, Carlsbad, CA, USA), and cDNA was reverse transcribed from total RNA using SuperScriptTM III First-Strand Synthesis System for real-time quantitative PCR (RT-PCR) (cat. no. 18080- 051, Invitrogen, Carlsbad, CA, USA) according to the procedures provided by the manufacturer. The expression levels of targeted genes involved in lipid synthesis and adipogenesis such as peroxisome proliferators activated receptor-gamma (PPAR-γ), CCAAT enhancer binding protein-alpha (C/EBP-α) and fatty acid binding protein 4 (FABP4), fatty acid synthase (Fasn), acetyl coenzyme A carboxylase 1 (Acc1), hydroxymethylglutaryl-CoA reductase (HMG-Red1) and sterol regulatory element binding protein-1c (SREBP-1c), and genes associated with fatty acid oxidation such as peroxisome proliferators activated receptor-alpha (PPAR-α) and the uncoupling protein 2 (UCP2), were measured using RT-PCR (CFX-96, Bio-Rad, USA) with β-actin as the invariant internal control. All the assays were performed in triplicate and normalized to the internal standard mRNA levels using the 2^−ΔΔCT^ method.

The oligonucleotide primers for the targeted genes in this study were designed and tested for efficiency using Primer-Blast as follows: PPAR-γ: F-5′TCGCTGATGCACTGCCTATG, R-5′GAGAGGTCCACAGAGCTGATT; C/EBP-α: F5′CAAGAACAGCAACGAGTACCG, R5′GTCACTGGTCAACTCCAGCAC; FABP4: F5′ AAGGTGAAGAGCATCATAACCCT, R5′TCACGCCTTTCATAACACATTCC; UCP2: F-5′ATGGTTGGTTTCAAGGCCACA3′, R-5′CGGTATCCAGAGGGAAAGTGAT3′; Acc1: F-5′CGAAGGGCTTACATTGCCTA3′, R-5′GGATGTTCCCTCTGTTTGGA3′; PPAR-α: F-5′AGAGCCCCATCTGTCCTCTC3′, R-5′ACTGGTAGTCTGCAAAACCAAA3′; SREBP-1c: F-5′TGACCCGGCTATTCCGTGA3′, R-5′CTGGGCTGAGCAATACAGTTC3′; Fasn: F-5′GGAGGTGGTGATAGCCGGTAT3′, R-5′TGGGTAATCCATAGAGCCCAG3′; HMG-red1: F-5′CTGGAATTATGAGTGCCCCAAA3′, R-5′ACGACTGTACTGAAGACAAAGC3′; β-actin: F-5′GGCCAACCGTGAAAAGATGA3′, R-5′CAGCCTGGATGGCTACGTACA3′.

### Statistical analysis

SPSS 17.0 was used to make statistical analysis in this study. One-way analysis of variance (ANOVA) was performed to compare the means of indexes among different groups with normally distributed data. The differences between these groups in data with the non-normal distribution were assessed using Mann–Whitney U-test and Wilcoxon signed-rank test. The Pearson correlation was used to determine the correlations between calcium and lipids (TG, TC) in the plasma and liver. All values were expressed as the means ± SD, and *P* < 0.05 was considered to be statistically significant.
